# Workplace Violence towards Healthcare Workers: An Italian Cross-Sectional Survey

**DOI:** 10.3390/nursrep11040072

**Published:** 2021-09-30

**Authors:** Nicola Ielapi, Michele Andreucci, Umberto Marcello Bracale, Davide Costa, Egidio Bevacqua, Nicola Giannotta, Sabrina Mellace, Gianluca Buffone, Vito Cerabona, Franco Arturi, Michele Provenzano, Raffaele Serra

**Affiliations:** 1 Department of Public Health and Infectious Disease, “Sapienza” University of Rome, 00185 Rome, Italy; vito.cerabona@uniroma1.it; 2 Interuniversity Center of Phlebolymphology (CIFL), International Research and Educational Program in Clinical and Experimental Biotechnology, University Magna Graecia of Catanzaro, Viale Europa, 88100 Catanzaro, Italy; davidcosta3@libero.it (D.C.); egidiobevacqua@unicz.it (E.B.); 3 Department of Health Sciences, University of Catanzaro, 88100 Catanzaro, Italy; andreucci@unicz.it; 4 Department of Public Health, University of Naples “Federico II”, 80138 Naples, Italy; umbertomarcello.bracale@unina.it; 5 Department of Law, Economics and Sociology, University Magna Graecia of Catanzaro, 88100 Catanzaro, Italy; 6 Department of Medical and Surgical Sciences, University of Catanzaro, 88100 Catanzaro, Italy; nicola.giannotta@studenti.unicz.it (N.G.); arturi@unicz.it (F.A.); michiprov@hotmail.it (M.P.); 7 Department of Surgery, Health Agency of Trento, 38100 Trento, Italy; mellace.sabrina@gmail.com; 8 Department of Vascular Surgery, Health Agency of Trento, 38100 Trento, Italy; gianlucabuffone@gmail.com

**Keywords:** workplace violence, healthcare workers, nurses

## Abstract

Background. Workplace violence (WPV) is a major healthcare problem with important consequences in healthcare areas and may impact negatively not only healthcare workers but also the quality and safety of patient care. Objectives: This an observational online web-based survey using Google^®^ Modules, specifically aiming to investigate the phenomenon of WPV in Italian healthcare services. Methods. Data collection for this study lasted one month, with the questionnaire available from 1 May 2021 to 31 May 2021. Continuous variables were considered as either mean ± standard deviation (SD) or median and interquartile range (IQR) based on their distribution. Comparison between groups was assessed by unpaired t-test or Mann–Whitney U test according to variable distribution. Categorical variables were analyzed using the chi-squared test. Results. The study population consisted of 203 healthcare workers, represented by nurses (61.6%), medical doctors (16.8%), patient care assistants (4.9%), and others (16.7%). Female gender was associated with a 2.6 times higher risk for the presence of aggression (*p* = 0.034), and nurse as a job with about 4 times increased risk for the presence of aggression (*p* = 0.006). The risk for aggression increased by 5% for each year of work experience. Conclusions. WPV is still matter of concern in Italian healthcare services. A strong organizational effort is demanded from healthcare institutions in order prevent internal and external violence in healthcare settings.

## 1. Background

Workplace violence (WPV) is related to the abuse, coercion, or assault of workers in any circumstances related to their work [1,2] and can be classified into two main types, as defined by the International Labour Organization: internal and external according to the perpetrators [3,4]. Specifically, the internal violence takes place between healthcare workers colleagues, supervisors, and managers, while the external violence takes place between healthcare workers and patients and their visitors (relatives, friends, etc.) [3].

The prevalence of WPV in health settings is not completely investigated as this phenomenon is often underreported due to the perception among healthcare workers that aggression and violence may be possible during their work activities, and also because of fear for the eventual personal consequences they may receive when reporting these events [1,5,6].

WPV is a major healthcare problem and can have important consequences both for healthcare staff and organizations as it is associated with work-related illness, job dissatisfaction, absenteeism, and also may affect the quality and safety of patient care [7,8,9].

## 2. Study

### 2.1. Aim

The aim of this study was to explore the WPV phenomenon among Italian healthcare workers.

### 2.2. Study Design and Procedures

We performed an observational online web-based survey using Google^®^ Modules, structured as in a previous work of our research group [10], specifically aiming to investigate the WPV phenomenon among healthcare workers in Italian healthcare settings. Data collection for this study lasted one month, with the questionnaire available from 1 May 2021 to 31 May 2021.

Participants were contacted through their available digital tools, namely, e-mail, WhatsApp contacts, social networks (Facebook), and the Google form was shared. Aggression was detected via the individual perception of verbal/psychological aggression during work or direct experience of physical aggression. All subjects who responded to our contact have been included in the analysis.

From a methodological point of view, a quantitative approach was adopted. Specifically, a survey with a questionnaire was used. It consisted of 43 requests with multiple answers. No scaling techniques were used to avoid the statistical and methodological problem with almost cardinal variables. In detail, there were three parts:

1. In the first part, there was the registry section (age, gender, education, working position, etc.).
2. In the second part, the three dimensions of violence were detected: verbal, psychological, and physical.
3. The third part showed the variables inherent to the health context.

Regarding the temporal dimension of violence, it should be noted that the episode of violence refers to any moment of the working period.

### 2.3. Ethical Considerations

The study was approved by the Institutional Review Board (IRB) of CIFL (approval number: E.R.ALL.2018.44.A), and all the healthcare workers who participated to the survey gave online informed consent.

### 2.4. Statistical Analysis

Continuous variables were reported as either mean ± standard deviation (SD) or median and interquartile range (IQR) based on their distribution. Comparison between groups was assessed by unpaired t-test or Mann–Whitney U test according to variable distribution. Categorical variables were analyzed using the chi-squared test. For the model building process, univariate analysis testing the association between the main variables and aggression, modeled as categorical variable (yes vs. no) was assessed by means of logistic regression analysis. The variables with *p* < 0.100 at univariate analysis were selected and included in the first multivariate logistic regression model. Next, a backward variable selection method with an elimination criterion of *p* ≤ 0.05 was performed to fit the final multivariate logistic regression model. Multicollinearity was assessed with variance inflation factors (VIF), which is a measure of the degree to which a single predictor variable can be expressed as a linear combination of the remaining predictor variables; values greater than 10 were cause for concern [11]. Data were analyzed using Stata version 16 (StataCorp, College Station, TX, USA).

## 3. Results

**Figure 1:**
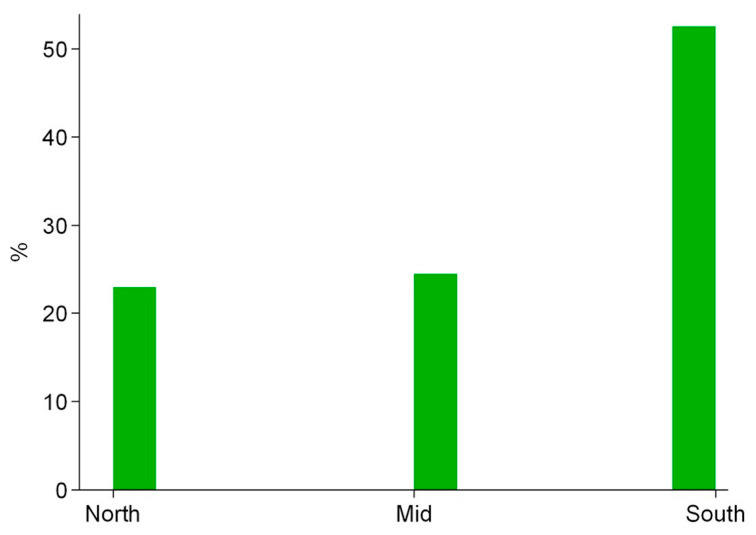
Distribution of working region for the subjects enrolled in our study.

The whole study population consisted of 203 healthcare workers, mainly represented by nurses (61.6%), medical doctors (16.8%), patient care assistants (4.9%), and others (16.7%). Overall, participating subjects were recruited from all the national territories with a homogeneous recruitment rate for north (23%) and middle (24.5%) Italy and with a larger rate for south (52.5%) Italy (Figure 1).

The overall cohort was characterized by young age (40.5 ± 10.8 years) and by a prevalence of women (122/203, 60.1%) over men (81/203, 39.9%) workers (Table 1).

Education level was good, as a large proportion of study subjects were graduated. Bachelor’s degree, as a title, had been earned more by women than men (*p* = 0.019), whereas men were more likely to achieve school of specialty (*p* = 0.025). Women were also more likely nurses than men (*p* = 0.020), while men were classified more frequently as medical doctors and patient care assistants (*p* = 0.037 and *p* = 0.046, respectively). Overall, prevalence of aggression among healthcare workers was striking, with 88.2% the frequency of verbal aggression, higher than the 60% and 30% frequencies of psychological and physical aggression, respectively (Table 1). Verbal aggression was more reported in women than in men (92.6% vs. 81.5%, *p* = 0.016), whereas prevalence of physical aggression was higher in men than in women (38.3% vs. 27.9%, *p* = 0.020). With respect to the source of violence, verbal aggression was mainly received by patients and relatives, psychological aggression by relatives being more frequent in men than in women (*p* = 0.009) in this subgroup, and physical aggression by relatives and patients.

When the variables with a strong association (*p* < 0.05) with aggression have been included in the multivariable model, after the stepwise selection, being female, a nurse, or an employee with longer time of work were significant correlates of the presence of aggression events (Table 2).

In particular, being female gender was associated with a 2.6 times higher risk for the presence of aggression (*p* = 0.034), and nurse as a job with about 4 times increased risk for the presence of aggression (*p* = 0.006). The risk for aggression increased by 5% for each year of work experience. The variance inflation factor was lower than 5 for all variables. Thus, we excluded collinearity with great confidence.

Moreover, no differences between the WPV phenomenon and private or public working settings was detected.

## 4. Discussion

Workplace violence in the healthcare area represents about a quarter of total violence events reported in all workplaces, with nurses being the most involved among healthcare workers [5,8].

In our study, nurses have an increased risk of suffering aggression, 4-fold greater than other healthcare professionals, and this can be due to the length of time spent with patients, and also a misperceived sense of authority compared, for example, with doctors [5].

In our study, female healthcare workers were more often assaulted compared with males, despite their professional category and this is in line with the current evidence worldwide [5,12,13].

Our study documented both internal and external aggression. Internal aggression is considered the most distressing form of WPV as bullying and harassment in the workplace coming from colleagues and managers has a particular negative impact on a health professional’s emotional health [7].

High rates of verbal aggression, both as result of internal and external violence, were reported in our study, followed also by psychological and physical aggression.

In the Italian context, Ferri et al. showed that 45% of healthcare professionals reported WPV [5]; Magnavita et al. reported an annual rate of WPV of 36.4% [8]. Our study reported a higher prevalence of aggression, up to 88.2% compared to those studies.

WPV has important and negative consequences for the worker, such as job disinterest, low productivity, drug abuse, alcohol abuse, burnout syndrome, depression and anxiety, possible suicidal ideation, and sensation of life dissatisfaction with consequent reduced overall quality of life [8,14].

If WPV affects the mental health of nurses and stress builds up in their thoughts, they may care for their patients with a sense of inadequacy and frustration, and this may adversely affect the subsequent quality of care for patients [9,15,16].

Experiencing WPV has been associated with a higher rate of burnout syndrome, which is a psychological alteration related to job activities particularly frequent among healthcare workers. WPV may also result in the development of posttraumatic stress disorder (PTSD) with depression, anxiety, and insomnia [14,17,18]. WPV may also result in the alteration of the secretion of the stress hormone cortisol that is dysregulated in PTSD patients [19].

Considering also the three dimensions of aggression (physical, psychological, and verbal), this study clearly demonstrates the multidimensional nature of the WPV phenomenon because it is the most evident manifestation of the so-called intergroup conflicts [20].

In our study, no differences between public or private working settings were found considering all aspects of WPV.

Therefore, the findings of this study allow us to affirm that there is a strong contrast between health professionals and patients, and between healthcare workers’ colleagues, supervisors, and managers, in the context of inadequate legal protection from WPV.

There are some limitations in this study: our findings have an observational nature due to the cross-sectional study design; the online web-based survey may lead to selection bias and limit the generalizability of the study; the assessment of WPV was based on self-reporting using the online tool and the subjects may perceive in different ways the items that were investigated; only subjects still working were included and this may lead to a selection bias as many subjects who experience WPV tend to abandon the workplace.

## 5. Conclusions

Our findings show that WPV is of major concern in healthcare daily activities coming from both internal and external contexts. A strong organizational effort is demanded from healthcare institutions to establish effective intervention strategies to predict and prevent aggression in healthcare settings and to better manage the actual cases of violence to prevent and treat the health consequences suffered by workers who experience WPV.

## Figures and Tables

**Table 1:** Basal characteristics of subjects enrolled in the study, overall and by gender.

Variables	Overall (n = 203)	Men (n = 81)	Women (n = 122)	*p*
Age, years	40.5 ± 10.8	41.4 ± 11.3	40.0 ± 10.5	0.351
Education, %				
Bachelor’s Degree	79.7	71.6	85.1	0.019
Master’s Degree	30.7	37.0	26.5	0.110
School of Specialty	16.3	23.5	11.6	0.025
PhD	3.0	3.7	2.5	0.615
Freelance workers, %	8.9	7.4	9.8	0.551
Temporary workers, %	9.9	11.1	9.0	0.624
Full-time workers, %	81.3	81.5	81.2	0.952
Job, n (%)				
Medical doctor	34 (16.7)	19 (23.5)	15 (12.3)	0.037
Nurse	125 (61.7)	42 (51.9)	83 (68.0)	0.020
Patient care assistant	10 (4.9)	7 (8.6)	3 (2.5)	0.046
Others	34 (16.7)	13 (16.0)	21 (17.2)	0.828
Working Region, %				0.792
North Italy	23.0	24.7	21.9	
Middle Italy	24.5	22.2	26.0	
South Italy	52.5	53.1	52.1	
Time of work, years	11 (5–21)	10 (5–21)	12 (5–21)	0.445
Verbal aggression, %	88.2	81.5	92.6	0.016
Source of verbal aggression, %				
Patient	54.2	53.1	54.9	0.798
Colleague	24.6	30.9	20.5	0.093
Relative	55.2	53.1	56.6	0.626
Man	63.6	61.7	64.8	0.661
Woman	49.8	49.4	50.0	0.931
Psychological aggression, %	64.0	64.2	63.9	0.969
Source of psychological aggression, %				
Patient	33.5	39.5	29.5	0.143
Colleague	21.7	24.7	19.7	0.314
Relative	37.9	49.4	30.3	0.009
Man	57.6	63.0	54.1	0.211
Woman	34.5	34.6	34.4	0.983
Physical aggression, %	32.0	38.3	27.9	0.020
Source of physical aggression, %				
Patient	23.2	24.7	22.1	0.672
Colleague	4.9	8.6	2.5	0.046
Relative	9.4	13.6	6.6	0.033
Man	28.6	35.8	23.7	0.043
Woman	12.8	12.4	13.1	0.872
Severity of aggression, %				
Very mild	5.4	6.2	4.9	0.699
Mild	3.5	6.2	1.6	0.083
Severe	2.0	1.2	2.5	0.539
Site of aggression, %				
Public structure	52.7	46.9	56.6	0.178
Private structure	4.4	6.2	3.3	0.327
Emergency department	16.3	23.5	11.5	0.023

**Table 2:** Logistic regression on the correlates of aggression in the study subjects.

Variables	Odds Ratio (n = 203)	95% (CI) (n = 81)	*p*
Gender, female vs. male	2.59	1.09–5.81	0.034
Time of work, for 1 year	1.05	1.00–1.10	0.046
Job, nurse vs. others	3.90	1.47–10.38	0.006

## Data Availability

The data presented in this study are available on request from the corresponding author. The data are not publicly available because an electronic link to the data has not been created.
